# Site-specific phosphorylation of Fbxw7 by Cdk5/p25 and its resulting decreased stability are linked to glutamate-induced excitotoxicity

**DOI:** 10.1038/s41419-019-1818-4

**Published:** 2019-08-02

**Authors:** Yeon Uk Ko, Chiho Kim, Juhyung Lee, Dana Kim, Yoonkyung Kim, Nuri Yun, Young J. Oh

**Affiliations:** 1 Department of Systems Biology, Yonsei University College of Life Science and Biotechnology, Seoul, 03722 Korea; 2 Department of Biochemistry, University of Texas Southwestern Medical Center, Dallas, TX 75390 USA; 3 Laboratory of Molecular Biology, National Institute of Diabetes and Digestive and Kidney Disease, National Institute of Health, Bethesda, MD 20892 USA; 4 Institute of Life Science & Biotechnology, Yonsei University, Seoul, 03722 Republic of Korea

**Keywords:** Cell death in the nervous system, Neurological disorders

## Abstract

Cyclin-dependent kinase 5 (Cdk5) is a serine/threonine protein kinase that regulates brain development and neurodegeneration. Cdk5 is activated by p25 that is generated from calpain-dependent cleavage of p35. The generation of p25 is responsible for the aberrant hyper-activation of Cdk5, which causes neurodegeneration. Using in vitro assays, we discovered that F-box/WD repeat-containing protein 7 (Fbxw7) is a new substrate of Cdk5. Additionally, Cdk5-dependent phosphorylation of Fbxw7 was detected in the presence of p25, and two amino acid residues (S349 and S372) were determined to be major phosphorylation sites. This phosphorylation was eventually linked to decreased stability of Fbxw7. Using a culture model of cortical neurons challenged with glutamate, we confirmed that decreased stability of Fbxw7 was indeed Cdk5-dependent. Furthermore, diminished levels of Fbxw7 led to increased levels of transcription factor AP-1 (c-Jun), a known substrate of Fbxw7. Given that previous reports demonstrate that c-Jun plays a role in accelerating neuronal apoptosis in these pathological models, our data support the concepts of a molecular cascade in which Cdk5-mediated phosphorylation of Fbxw7 negatively regulates Fbxw7 expression, thereby contributing to neuronal cell death following glutamate-mediated excitotoxicity.

## Introduction

Cyclin-dependent kinases (CDKs) comprise a family of protein kinases that regulates the cell cycle^1,2^. One member of the CDK family, Cdk5, is primarily activated in the brain and plays a role in brain development, neuronal maturation, and synaptic plasticity^3,4^. This member has a unique physiological characteristic compared to other CDKs^5^. Specifically, Cdk5 is activated by neuron-specific regulatory proteins p35 or p39 instead of cyclin^6,7^. This distinctive property allows Cdk5 to play a unique role in neuronal cell physiology. Cdk5 phosphorylates specific substrates that are involved in axonal and dendritic growth as well as neuronal migration. In contrast to these beneficial effects, aberrant regulation of Cdk5 activity causes neurodegenerative disease^8^. Notably, the cleavage of p35 into p25 by calpain, a calcium-activated cysteine protease, leads to Cdk5 hyper-activation in response to neurotoxin treatment^9^. Under this condition, for example, Cdk5/p25 acts as a regulator of cell death by phosphorylating tau and p53^10,11^. Therefore, discovery of a novel Cdk5 substrate is necessary to expand our understanding of the primary regulating function of Cdk5/p25 in neuronal death.

F-box/WD repeat-containing protein 7 (Fbxw7) is a substrate recognition component of the SKP1–Cul1–F-box protein (SCF^FBXW7^) E3 ligase complex that regulates degradation of proteins involved in cell division and growth^12,13^. Fbxw7 is associated with tumor suppression because most of its substrates are proto-oncogene proteins, including c-Jun^14^, cyclin E^15^, c-Myc^16^, Notch^17^, and Mcl-1^18^. Most Fbxw7 substrates contain Cdc4 degron motifs phosphorylated by glycogen synthase kinase 3^19^. Downregulation or mutation of Fbxw7 leads to the development of various cancers in humans. Many Fbxw7-related studies have therefore focused primarily on understanding of its role in cancer metabolism. Interestingly, Fbxw7 has also been reported to play an important role during brain development and differentiation by regulating Notch and c-Jun^20,21^. Since the publication of a report demonstrating the importance of maintaining the stability of Fbxw7 to retain its biological function^22^, several studies have focused on determining upstream molecules that directly govern Fbxw7 expression and degradation; for examples, polo-like kinase 2 (Plk2) phosphorylates and regulates the degradation of Fbxw7^23^, and COP9 signalosome subunit 6 (CSN6) promotes ubiquitination and degradation of Fbxw7^24^. However, despite the widespread expression of Fbxw7 in the brain, its regulatory mechanism and pathophysiological role during neurodegeneration have been less studied.

The aim of this study was to explore the mechanism that governs the stability and expression levels of Fbxw7. We used a neuronal culture model of excitotoxicity to determine if calpain-mediated cleavage of p35 to p25, which is indicative of Cdk5 hyper-activation^25^, was evident in this model and if this event was linked to decreased expression levels of Fbxw7. We also investigated the correlation of expression levels between Fbxw7 and its well-known cellular substrate, c-Jun. We hypothesized that regulation of Fbxw7 is important to maintain neuronal cell homeostasis and that the phosphorylation status of Fbxw7 may act as a critical player in Cdk5-mediated neuronal death.

## Results

### Cdk5/p25 physically interacts with and phosphorylates Fbxw7

**Fig. 1:**
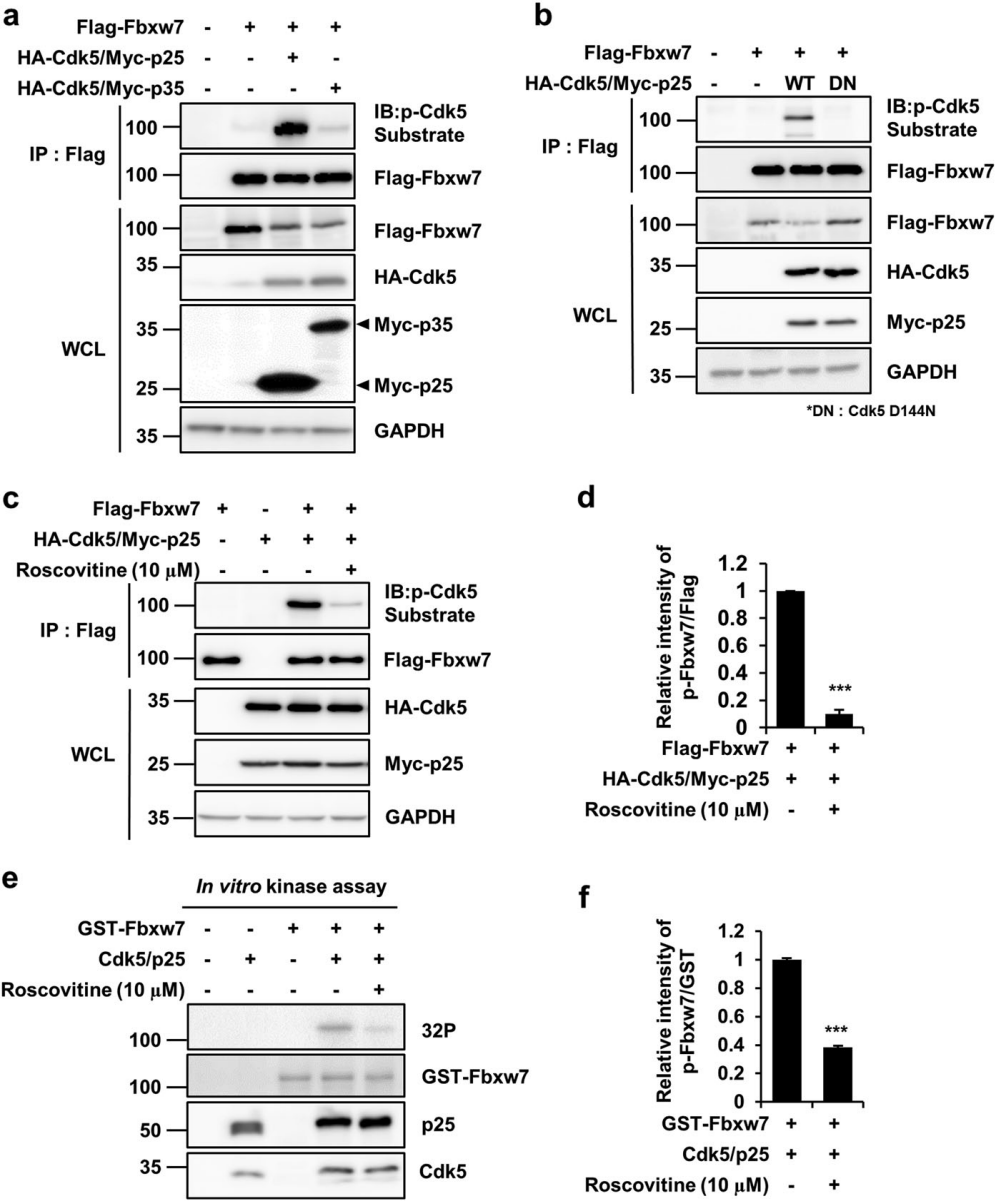
Cdk5/p25 phosphorylates Fbxw7. **a** HEK293 cells were transfected with Flag-Fbxw7 in combination with either HA-Cdk5/Myc-p25, HA-Cdk5/Myc-p35, or neither for the control. Cell lysates were incubated with Flag-conjugated beads for immunoprecipitation (IP). Immunoprecipitates and whole cell lysates (WCL) were resolved by SDS-PAGE and followed by immunoblot analysis (IB). Phospho-Fbxw7 signal was visualized with an anti-phospho-Cdk5-substrate antibody. GAPDH was used as a loading control. **b** HEK293 cells were transfected with Flag-Fbxw7 in combination with either HA-Cdk5 WT/Myc-p25 or HA-Cdk5 dominant-negative D144N (DN)/Myc-p25. WCL were used to IP using Flag-conjugated beads and IB analysis was done using the indicated antibodies. **c** HEK293 cells were exposed to 10 μM roscovitine for 48 h. Immunoprecipitates were purified with an anti-Flag antibody and IB was conducted with the indicated antibodies. **d** After normalization to Flag-Fbxw7, the relative fold intensity of the phospho-Fbxw7 signal by Cdk5/p25 were measured in samples treated with or without roscovitine (value = 1). Bar represents the mean ± SD of three independent experiments. ***p < 0.001. **e** Purified GST-Fbxw7 protein (1 µg) was incubated with or without 0.1 µg recombinant His-Cdk5/GST-p25 protein in the presence of [γ-32P] ATP. Reaction mixtures were resolved by SDS-PAGE and subjected to autoradiography. **f** After normalization to GST-Fbxw7, the relative fold intensity of the phospho-Fbxw7 signal by Cdk5/p25 was measured in samples treated with or without roscovitine (value = 1). The bar represents the mean ± SD of three independent experiments. ^***^p < 0.001

Phosphorylation of Fbxw7 by Cdk5, induced by transient transfection of Fbxw7 and Cdk5 with p25 or p35 activator into human embryonic kidney 293 (HEK293) cells, was observed in the presence of Cdk5/p25 (Fig. 1a) and to a marginal degree in the presence of Cdk5/p35 (Supplementary Fig. 1a, b). This result could be due to the cellular localization of Fbxw7, which has been reported to be primarily in the nucleus and cellular vesicle^22^, whereas p35 is mainly localized in the plasma membrane by myristolylation^26^. Moreover, this discrepancy may be ascribed to a rapid turnover of Cdk5/p35 as compared to that of Cdk5/p25^27^. To determine whether phosphorylation of Fbxw7 is dependent on Cdk5 kinase activity, we transfected the Cdk5-dominant-negative mutant (Cdk5 DN) into HEK293 cells and subsequently analyzed Fbxw7 phosphorylation levels. Phosphorylation of Fbxw7 was completely diminished in combination with Cdk5 DN compared to Cdk5 wild-type (Cdk5 WT) (Fig. 1b). Similarly, the Fbxw7 phosphorylation was dramatically decreased after treatment with Cdk5 inhibitor, roscovitine. (Fig. 1c, d). Direct phosphorylation of Fbxw7 by Cdk5/p25 was confirmed by in vitro assay, and Fbxw7 phosphorylation was again found to be reduced after addition of roscovitine (Fig. 1e, f).

**Fig. 2:**
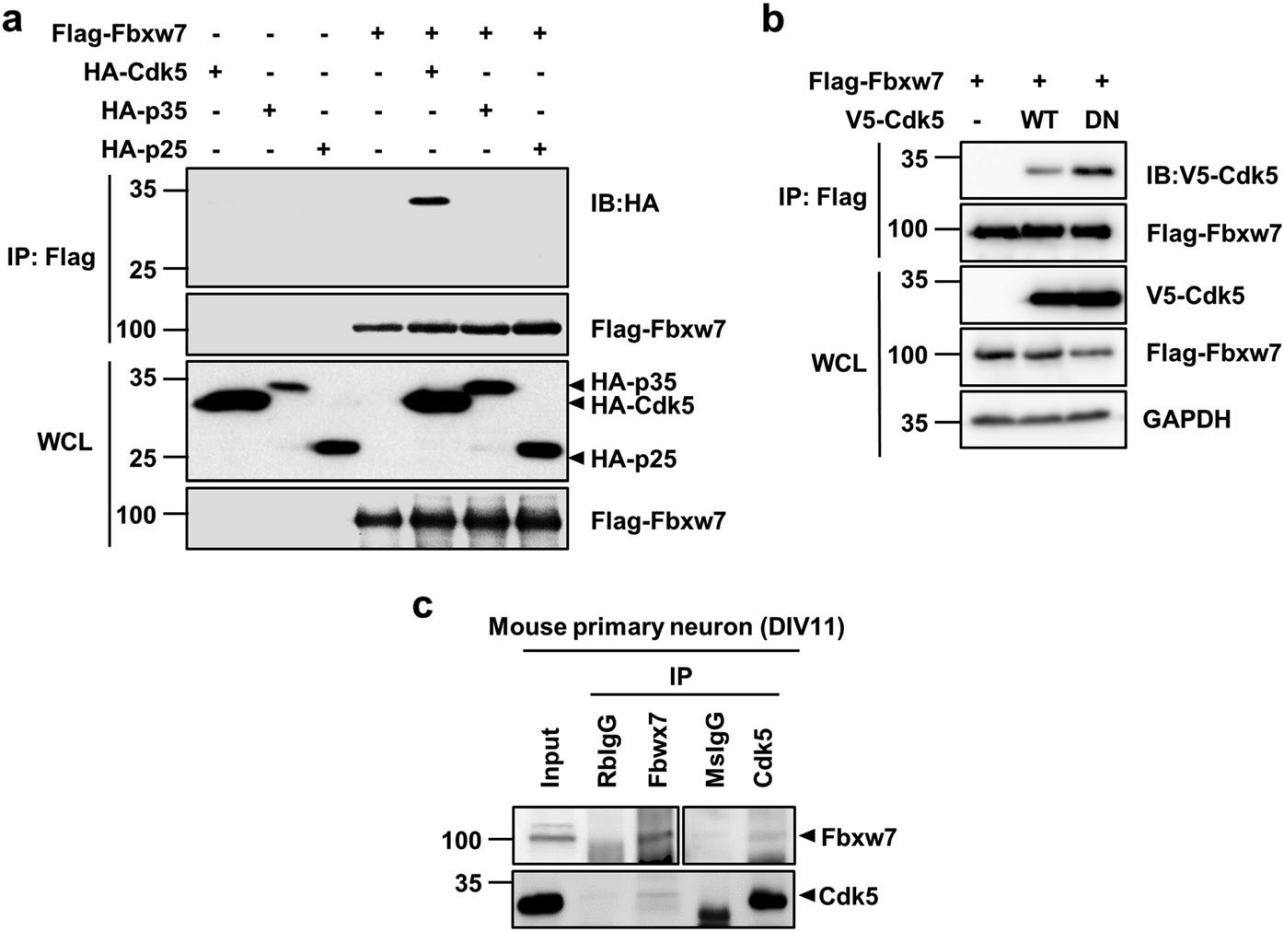
Cdk5 interacts with Fbxw7. **a** Following transfection of HEK293 cells with the indicated combination of vectors, cell lysates were subjected to IP using Flag-conjugated beads. Immunoprecipitates and WCL were resolved by SDS-PAGE and analyzed by IB analysis. **b** HEK293 cells were transfected with Flag-Fbxw7 alone or in combination with either V5-Cdk5 WT/Myc-p25 or V5-Cdk5 DN/Myc-p25. IP followed by IB was performed as described above. **c** Primary cultures of cortical neurons were maintained until days in vitro(DIV) 11. IP was performed with anti-Fbxw7 or anti-Cdk5 antibodies. Rabbit anti-IgG and mouse anti-IgG antibodies were used as IP controls. Fbxw7 was detected by IB after IP pulldown with anti-Cdk5 antibody and vice versa

Immunoprecipitation assay results for the physical interaction between Fbxw7 and Cdk5 in transfected HEK293 cells showed that Fbxw7 physically bonded to Cdk5, but not to p35 or p25 activators (Fig. 2a). Interestingly, Cdk5 DN also effectively bound to Fbxw7, indicating that the kinase activity of Cdk5 was not responsible for Cdk5 binding to Fbxw7 (Fig. 2b). Endogenous binding of these two proteins was confirmed in cell extracts obtained from primary cultures of cortical neurons (Fig. 2c). In summary, we found that Cdk5 physically interacts with and phosphorylates Fbxw7 in the presence of p25.

### Ser349 and Ser372 are the major Cdk5/p25 phosphorylation sites of Fbxw7

**Fig. 3:**
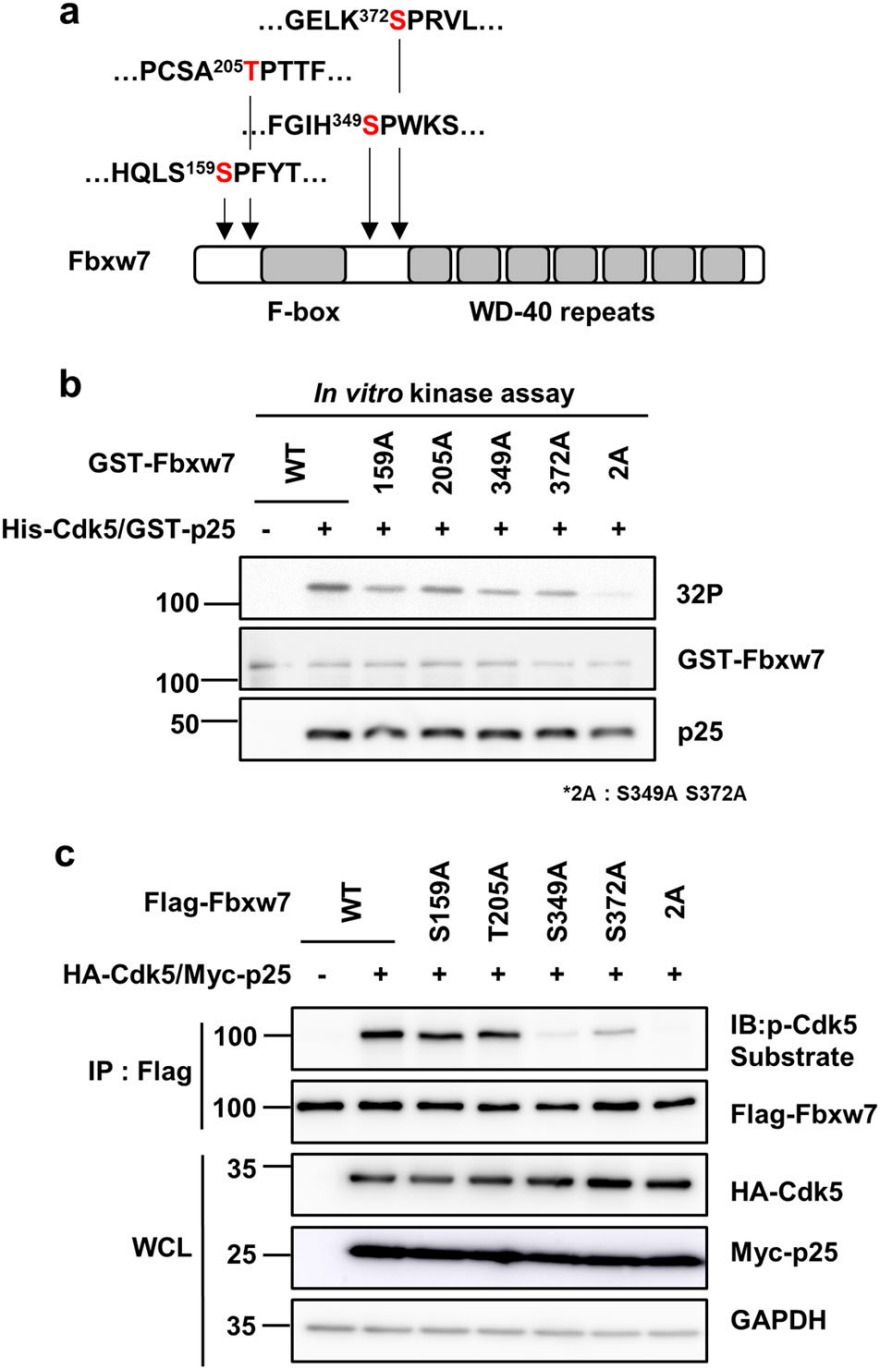
Both Ser349 and Ser372 are the major sites of Fbxw7 that are phosphorylated by Cdk5/p25. **a** Scheme for putative Cdk5-phosphorylaton sites of Fbxw7 (S/TPXK/R). **b** In vitro kinase assay to detect the direct phosphorylation sites of Fbxw7 by Cdk5/p25. Purified GST-Fbxw7 wild-type (WT) protein (1 µg) or one of the GST-Fbxw7 site-specific mutant proteins were incubated with 0.1 µg recombinant His-Cdk5/GST-p25 protein in the presence of [γ-32P] ATP. Reaction mixtures were resolved by SDS-PAGE and subjected to autoradiography. **c** HEK293 cells were transfected with Flag-Fbxw7 WT or one of the Flag-Fbxw7 site-specific mutant proteins in the presence or absence of HA-Cdk5/Myc-p25. For comparison with the p-Fbxw7 signal, HEK293 cells were transfected with Flag-Fbxw7 2A mutant plus HA-Cdk5/Myc-p25. Cell lysates were incubated with Flag-conjugated beads for IP. Immunoprecipitates and WCL were resolved by SDS-PAGE analyzed by IB analysis. Phospho-Fbxw7 signal was visualized with an anti-phospho-Cdk5-substrate antibody

Next, we explored the specific Cdk5/p25 phosphorylation site(s) of Fbxw7. Bioinformatics analysis revealed four putative (Ser/Thr)-Pro Cdk5 phosphorylation motifs of Fbxw7 (Fig. 3a). We generated various site-specific, phospho-null mutants of Fbxw7 to investigate the major phosphorylation site of Fbxw7 by Cdk5. The mutant proteins were incubated with Cdk5/p25 for an in vitro kinase assay. There was an overall decrease in the intensity of the phospho-Cdk5 signal in the samples with Fbxw7 single mutant isoforms compared to Fbxw7 WT (Fig. 3b). The decreased signal of Fbxw7 phosphorylation was much more prominent in the Fbxw7 double mutant (2A; S349A/S372A). At the cellular level, the phosphorylation signal of Fbxw7 was markedly decreased in the sample obtained from the S349A or S372A mutant. A more dramatic decrease of the phosphorylation signal was found in the Fbxw7 double mutant (2A; S349A/S372A, Fig. 3c). Unlike data obtained from the in vitro kinase assays, no discernible changes were detected in the S159A or T205A mutant samples as compared with Fbxw7 WT samples in the cell-based assay. Although we did not determine the reason for discrepancy in results between the in vitro kinase assay and the cell-based assay, one plausible explanation could be that the cellular localization of Fbxw7 restricted interaction with its specific partners. Taken together, our data indicate that Ser349 and Ser372 were the major Cdk5/p25 phosphorylation sites of Fbxw7.

### Cdk5/p25-mediated phosphorylation leads to decreased Fbxw7 stability

**Fig. 4:**
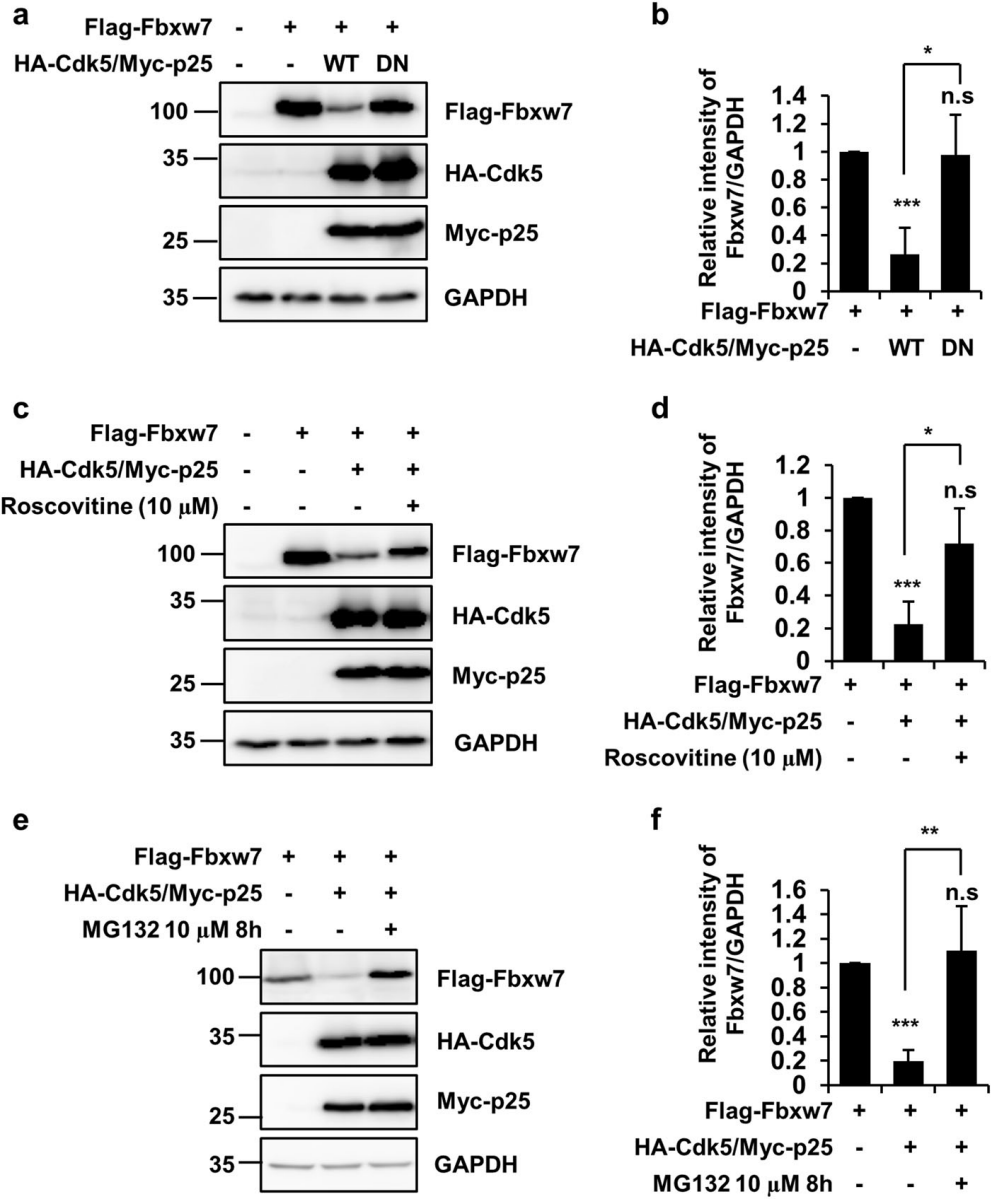
Cdk5 negatively regulates Fbxw7 protein expression level. **a** HEK293 cells were transfected with Flag-Fbxw7 alone or in combination with either HA-Cdk5 WT/Myc-p25 or HA-Cdk5 DN/Myc-p25. IB analysis was conducted using the indicated antibodies. **b** Relative intensity of Flag-Fbxw7 was calculated over the control (only transfected with Fbxw7; value = 1) after normalization against GAPDH. Bar represents the mean ± SD of three independent experiments. ^*^p < 0.05; ^***^p < 0.001; n.s not significant. **c** HEK293 cells were exposed to 10 μM roscovitine for 48 h. IB analysis was conducted with the indicated antibodies. **d** Relative intensity of Flag-Fbxw7 intensity was calculated over the control (value = 1) after normalization against GAPDH. Bar represents the mean ± SD of three independent experiments. ^*^p < 0.05; ^***^p < 0.001; n.s not significant. **e** HEK293 cells transfected with Flag-Fbxw7 alone or in combination with HA-Cdk5/Myc-p25 were treated with or without 10 μM MG132 for 8 h. Cell lysates were analyzed by IB using the indicated antibodies. **f** Relative intensity of Flag-Fbxw7 was calculated over the control (value = 1) after normalization against GAPDH. Bar represents the mean ± SD of three independent experiments. ^**^p < 0.01; ^***^p < 0.001; n.s not significant

It has been previously demonstrated that degradation of Fbxw7 is regulated by Plk2^23^ and CSN6^24^. For example, Plk2 phosphorylates Fbxw7 on Ser25, Ser176, and Ser349, and this event leads to Fbxw7 degradation. Therefore, we specifically investigated whether Cdk5/p25-mediated phosphorylation of Fbxw7 affected Fbxw7 stability. When HEK293 cells were co-transfected with Fbxw7 and Cdk5/p25, we observed decreased expression levels of Fbxw7 (Fig. 4a, b). In contrast, expression levels of Fbxw7 were not affected when HEK293 cells were co-transfected with Cdk5 DN mutant, indicating the necessity of Cdk5 kinase activity. Similarly, the Cdk5/p25-meidated decrease of Fbxw7 was inhibited in the presence of roscovitine (Fig. 4c, d). Based on a previous report demonstrating that Fbxw7 is ubiquitinated and degraded rapidly via an autocatalytic mechanism^28^, we tested whether Cdk5/p25-mediated phosphorylation of Fbxw7 results in Fbxw7 degradation via the ubiquitin-proteasome system. We showed that Cdk5/p25-dependent degradation of Fbxw7 was blocked in the presence of MG132 (Fig. 4e, f).

**Fig. 5:**
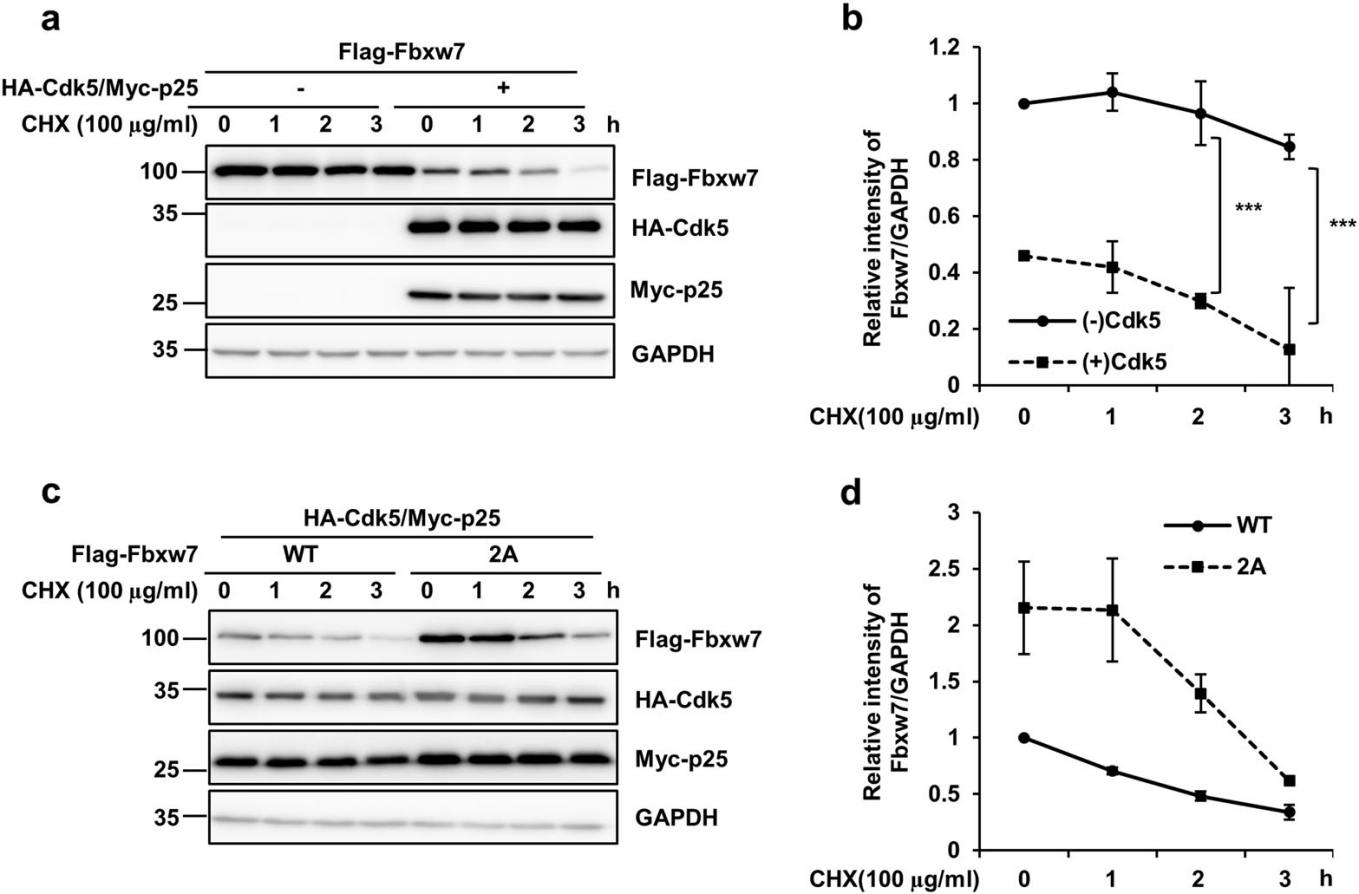
Cdk5-mediated phosphorylation causes the decreased stability of Fbxw7. **a** HEK293 cell were transfected with Flag-Fbxw7 WT alone or in combination with Cdk5/Myc p25. Forty-eight hour after transfection, cells were incubated in the presence of 100 μg/ml cycloheximide (CHX) for the indicated time periods. Cell lysates from each condition were analyzed by IB using the indicated antibodies. **b** After normalization against GAPDH, relative levels of Flag-Fbxw7 at the designated time periods were evaluated against time point 0 and shown in a graph. Bar represents the mean ± SD of three independent experiments. ^***^p < 0.001. **c** HEK293 cells were transfected with Flag-Fbxw7 WT or Flag-Fbxw7 2A plus HA-Cdk5/Myc-p25. Forty-eight hour after transfection, cells were incubated with 100 μg/ml CHX. Cell lysates were analyzed by IB using the indicated antibodies. **d** After normalization against GAPDH, relative levels of Flag-Fbxw7 at the designated time periods were evaluated against time point 0 and shown in a graph. Bar represents the mean ± SD of three independent experiments

We also performed cycloheximide (CHX) chase assays to confirm that site-specific phosphorylation of Fbxw7 by Cdk5 is linked to decreased stability of Fbxw7. When HEK293 cells were transfected with Fbxw7 and Cdk5/p25, the basal level of Fbxw7 was less than that in HEK293 cells transfected only with Fbxw7 (Fig. 5a, b). Furthermore, analysis of protein stability by the CHX chase assay indicated that the half-life of Fbxw7 was at ~2.5 h in HEK293 cells transfected with Fbxw7 and Cdk5/p25. In contrast, when HEK293 cells were transfected with only Fbxw7, significantly higher levels of Fbxw7 still remained at this time point. Moreover, HEK293 cells transfected with Cdk5 phospho-null mutant (Fbxw7 2A) exhibited increased levels of Fbxw7 at all the same time points as those in HEK 293 cells transfected with WT Fbxw7 (Fig. 5c, d). Altogether, our data indicate that Cdk5/p25 is another important negative regulator of Fbxw7 expression. In this study, we determined whether mRNA levels of Fbxw7 were affected in Cdk5-activating condition. We performed quantitative real-time reverse transcriptase-polymerase chain reaction (qRT-PCR) and compared the Fbxw7 mRNA expression of cells transfected with or without Cdk5/p25. We found that mRNA levels of Fbxw7 remained the same regardless of Cdk5 activation state (Supplementary fig. 2), indicating that Cdk5 regulates Fbwx7 level by the posttranslational modification.

### Hyper-activation of Cdk5 is responsible for decreased expression of Fbxw7 during glutamate-mediated excitotoxicity

**Fig. 6:**
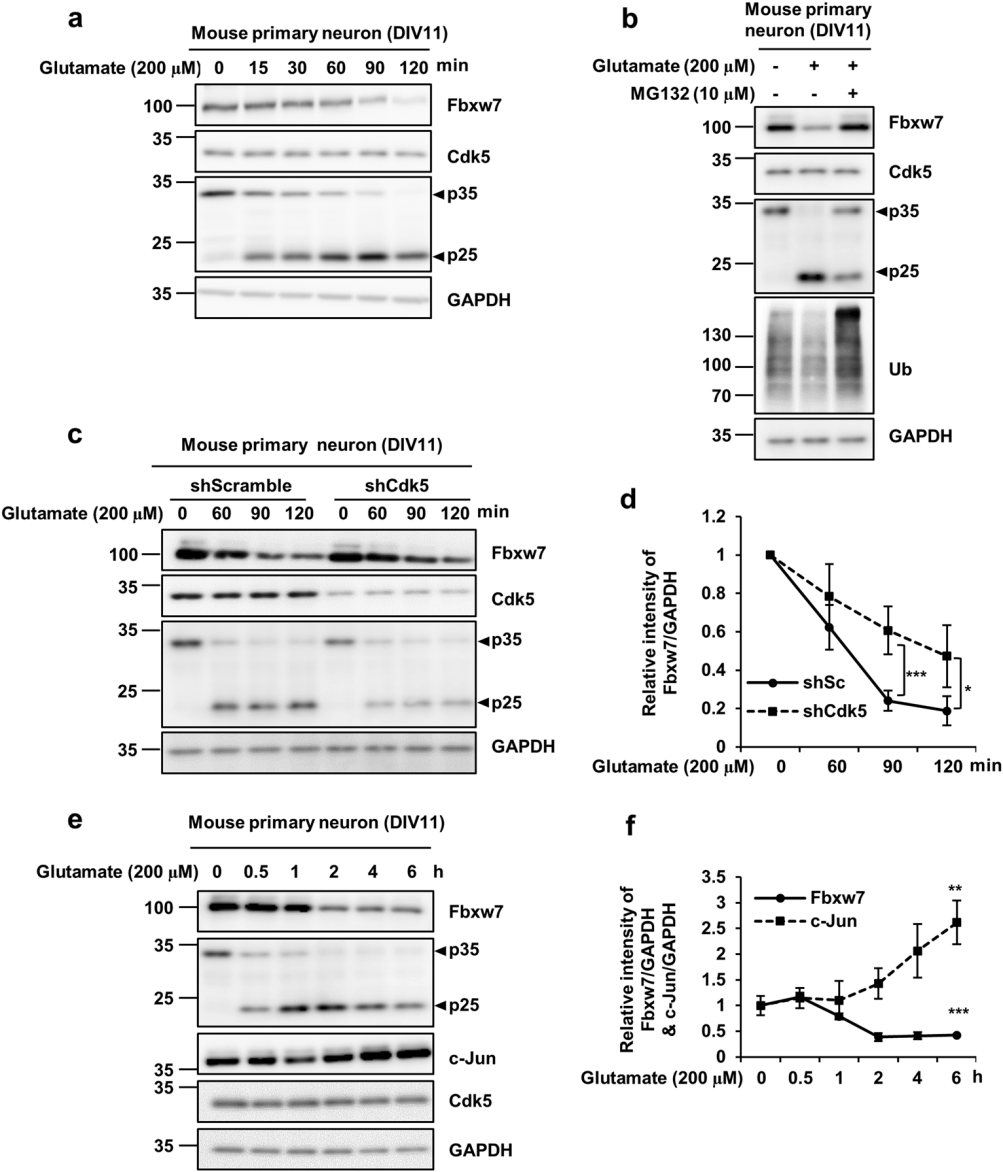
Inverse expression patterns of Fbxw7 and its substrate c-Jun detected in cortical neurons during glutamate excitotoxicity. **a** Primary cultures of cortical neurons were exposed to 200 μM glutamate for the indicated time periods or **b** cultures were exposed for 2 h to 200 μM glutamate in the presence or absence of 10 μM MG132. **a**, **b** Cell lysates were harvested and analyzed by IB. **c** Primary cultures of cortical neurons were infected with lentiviral particles containing shRNA against either Cdk5 or control (Scramble) at DIV3 and subsequently maintained until DIV 11. Cultures were then exposed to 200 μM glutamate for the indicated time periods. Cell lysates were subjected to IB analysis. **d** After normalization against GAPDH, relative levels of Fbxw7 at the designated time periods were evaluated against time point 0 and are shown in the graph. Each point represents the mean ± SD from five independent experiments. ^*^p < 0.05; ^***^p < 0.001^.^ **e** Primary cultures of cortical neurons were exposed to 200 μM glutamate for up to 6 h. Cell lysates were harvested and analyzed by IB. **f** After normalization against GAPDH, relative fold intensity of Fbxw7 and c-Jun signal was quantified over the control at time 0 (value = 1). Each point represents the mean ± SD from three independent experiments. ^**^p < 0.01; ^***^p < 0.001

It has been well established that dysregulated activation of Cdk5 is associated with neuronal death^4^. Mechanistically, calpain is activated by disruption of calcium homeostasis resulting from oxidative stress, which can be induced by a neurotoxin^29^. Activated calpain cleaves p35 to p25, ultimately leading to Cdk5 hyper-activation^8^. Therefore, we examined whether Cdk5 hyper-activation is linked to decreased expression of Fbxw7 in pathological models of excitotoxicity^30^. We chose a primary culture of cortical neurons that were treated with glutamate to induce Cdk5 hyper-activation and to subsequently determine endogenous levels of Fbxw7. As shown in Fig. 6a, expression levels of Fbxw7 were decreased over time in response to glutamate treatment. Interestingly, decreased levels of Fbxw7 coincided with calpain-mediated cleavage of p35 into p25. We further investigated whether these phenomena were recapitulated in other calpain-activating conditions in which cells were treated with hydrogen peroxide (Supplementary fig. 3a, c) or N-methyl-4-phenylpyridinium iodide (MPP^+^) (Supplementary fig. 3b, d). All neurotoxins dramatically reduced the levels of Fbxw7 at 120 min after exposure. This result was accompanied by p35 conversion into p25. Similar to the pattern found in HEK293 cells, glutamate-induced decrease in Fbxw7 was significantly restored in cortical neurons co-treated with MG132 (Fig. 6b). To unambiguously demonstrate that decreased expression of Fbxw7 is Cdk5-activity-dependent, we made a Cdk5-knockdown model using a lentivirus infection system as previously described in our lab^30^. The relative intensity of Fbxw7 was higher in Cdk5-knockdown cortical neurons than cortical neurons infected with lentivirus with short hairpin scramble (shScramble) (Fig. 6c, d). We next rationalized that decreased levels of Fbxw7 would result in the accumulation of its respective substrate, which would therefore be enriched. Indeed, it has been demonstrated that up-regulation of c-Jun is attributed to a decreased level of Fbxw7^20^. As expected, the level of c-Jun increased concomitantly with a decrease of Fbxw7 (Fig. 6e, f), confirming their inverse relationship. These data indicate that glutamate-induced excitotoxicity was accompanied by downregulation of Fbxw7 in a Cdk5-activity-dependent manner. This result was attributed to up-regulation of the pro-cell-death factor c-Jun. Therefore, we suggest that degradation of Fbxw7 and concomitant accumulation of c-Jun may play a critical role in regulating excitotoxicity that is accompanied by Cdk5 hyper-activation.

## Discussion

**Fig. 7:**
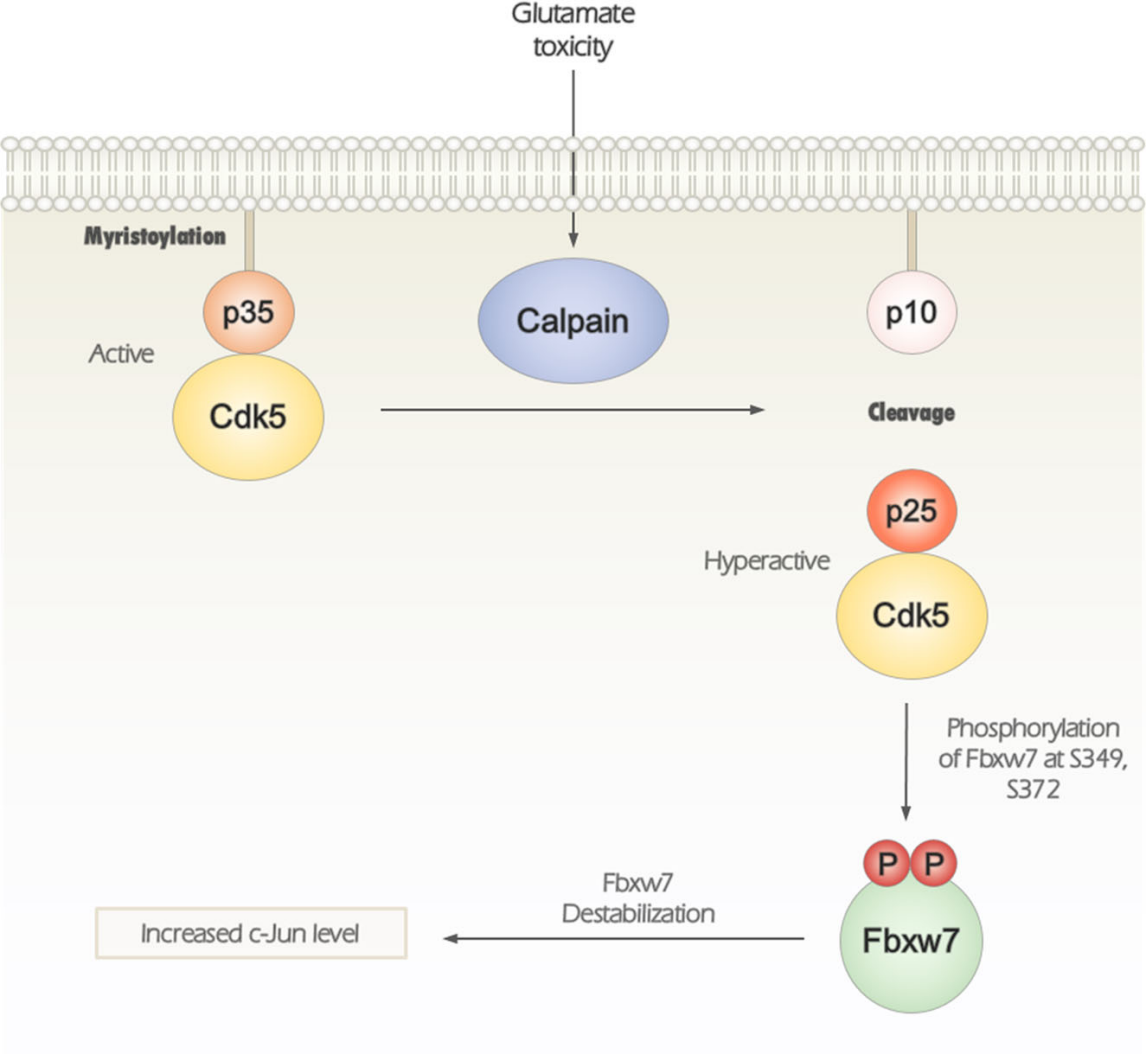
A schematic model of the consequence of Cdk5-mediated phosphorylation of Fbxw7. During glutamate-mediated excitotoxicity, activated calpain cleaves p35 into p25 leading to Cdk5 hyper-activation. Cdk5 in association with of activating cofactor p25 phosphorylates Fbxw7 at S349 and S372. Subsequently, Cdk5/p25-mediated phosphorylation induces destabilization of Fbxw7, resulting in a concomitant increase of its cellular substrate, c-Jun

Fbxw7 is a tumor suppressor that acts via regulation of cell growth, division, and differentiation. Its anti-cancer effects are carried out through degradation of several target onco-proteins, including c-Jun, Notch1, c-Myc, and cyclin E. Therefore, cellular regulation of Fbxw7 is an important step in maintaining cellular homeostasis. Although it has been reported that Fbxw7 is ubiquitously distributed in human tissues including the brain^31^, the regulation and functional consequence of Fbxw7 in neurons has been less examined. Here, we proposed that the novel mechanism underlying glutamate-mediated excitotoxicity, which is phosphorylation of Fbxw7 by hyper-activated Cdk5, may be a critical step to determine neuronal death. Specifically, we demonstrated that Fbxw7 is a novel substrate of Cdk5 and activated Cdk5 phosphorylates S349 and S372 residues of Fbxw7. We found that Cdk5-mediated phosphorylation of Fbxw7 is linked to destabilization of Fbxw7. Using a cortical neuronal culture model of excitotoxicity, we demonstrated an inverse relationship between expression of Fbxw7 and its substrate, c-Jun^14^. As such, accumulation of c-Jun was a consequence of its escaping from the Fbxw7-dependent degradation as a result of Cdk5-dependent decreased expression of Fbxw7. Recently, the phosphorylation of Fbxw7 was discovered^23,32^ and its decreased level was observed during oxidative stress^33^. As c-Jun is regarded as a pro-apoptotic protein^34^, we propose a temporal neuronal death pathway in which decreased expression of Fbxw7 via Cdk5 hyper-activation eventually leads to accumulation of c-Jun. This result may play an important cytotoxic role during excitotoxicity (Fig. 7).

Cdk5 is a well-known Ser/Thr kinase and a member of the highly conserved cyclin-dependent kinase family, but it is not involved in cell cycle progression. Although it is ubiquitously expressed, its co-activator proteins (p35 and p39) are exclusively expressed in neurons. Cdk5 kinase activity is prominently confined into the nervous system and, therefore, is involved in neuronal differentiation and neuronal death^3^. Consequently, appropriate regulation of Cdk5 activity is important to maintain neuronal homeostasis^6^. Like the conversion of p35 to p25^26^, dysregulated Cdk5 activity has been linked to an array of neurodegenerative diseases^8^. In these pathological conditions, hyper-activated Cdk5 phosphorylates a variety of cellular substrates, triggering a cascade of neurotoxic pathways that culminate in neuronal death. Efforts to identify several key substrates of Cdk5 have received significant attention. For example, it has been demonstrated that Cdk5 plays a key role in tau phosphorylation and tangle formation^11^. Therefore, RNA interference (RNAi) targeting of Cdk5 reduces tau phosphorylation and decreases the number of neurofibrillary tangles in the hippocampus of triple-transgenic Alzheimer’s disease (AD) mice^35^. Luo et al. demonstrated that Cdk5 phosphorylates huntingtin, and this phosphorylation reduces its cleavage by caspases and results in attenuation of aggregate formation and neurotoxicity^36^. Similarly, it has been shown that phosphorylation by Cdk5 decreases E3 ligase activity of Parkin and modulates the formation of inclusion bodies^37^, demonstrating the cases in which Cdk5-mediated phosphorylation of the disease-related proteins is involved in pathogenesis of several neurodegenerative diseases. On the other hand, accumulating evidence has indicated that Cdk5 acts as an important link between disease-initiating factors and cell-death effector^38^. Along this line of investigations, several recent studies, including reports from our laboratory, have raised the possibility that Cdk5-mediated phosphorylation of the E3 ligase complex is critically involved in pathogenesis^30,39,40^. In support of this notion, the present study provides an additional regulation of pro-apoptotic protein by Cdk5-mediated phosphorylation of E3 ligase component, in which accumulation of c-Jun is achieved by decreased expression and stability of the phosphorylated Fbxw7. Although we have not examined whether Cdk5 directly phosphorylates c-Jun^41^ and Mcl-1^42^ after excitotoxicity, our results support a notion that accumulation of c-Jun comprises a specific execution phase in the temporal cell-death sequences of excitotoxicity. In consideration of a previous report^42^ demonstrating that hyper-activated Cdk5 phosphorylates and triggers Mcl-1 ubiquitination after excitotoxicity, we propose that Cdk5/p25-mediated Fbxw7 destabilization could accelerate cell death via two pathways; namely, accumulation of pro-apoptotic c-Jun and destabilization of anti-apoptotic Mcl-1. Surge of intracellular free Ca^2+^ level caused by disturbance of Ca^2+^ homeostasis has been considered as one of the major changes that occurs following ischemic stroke. Interestingly, previous study has demonstrated that ischemic stroke injury is mediated by aberrant Cdk5 activation^25^. Consequently, they have demonstrated that pharmacological inhibition or conditional knockout of Cdk5 prevents neuronal death in response to ischemic injury. Based on this and other related studies, we speculate that ischemia-mediated activation of Cdk5 may lead to decreased expression of Fbxw7 and increased expression of c-Jun. Therefore, we infer that regulation of Cdk5 activity may be the potential therapeutic target for ischemic injury.

Here, we raised the possibility that the Cdk5-Fbxw7-c-Jun axis plays a critical role in promoting neuronal death. To date, dysfunction of E3 ligase and its associated ubiquitin-proteasome system (UPS) has been observed in neurodegenerative diseases and is an important therapeutic target^43,44^. Protein turnover is important to maintain homeostasis of post-mitotic neurons, and UPS plays a major role in controlling protein quality to ensure healthy neuronal function^45^. Many neurodegenerative diseases are characterized by the presence of aggregate-prone neurotoxic proteins that are believed to perturb neuronal function. Therefore, malfunction of the UPS has been believed to be one of the prime culprits for generation of abnormal protein aggregates^46^. In this regard, several E3 ligases have been implicated in the pathogenesis of neurodegenerative diseases. For example, decreased activity of Parkin found in an AD mouse model and overexpression of Parkin ameliorates beta-amyloid load and restores behavioral abnormalities^47^. Similarly, loss-of function mutations in Parkin is shown to impair its E3 ligase activity and leads to the accumulation of protease-resistant alpha-synuclein and the formation of Lewy bodies^48,49^. Dysfunction of the SCF complex has been indicated in the pathogenesis of poly-glutamine diseases like Huntington’s disease (HD)^50^. Also, reduced levels of Cul1 and Skp1 were found in HD mice brain and the cellular HD model. Consequently, silencing of Cul1 results in increased aggregate load and enhanced poly-glutamine-induced neurotoxicity, arguing that reduced levels of SCF complex contribute to the pathogenesis of poly-glutamine disease. Fbxw7, a well-characterized SCF component, contributes to substrate specificity and mediates ubiquitination of proteins targeted for degradation by the UPS. Therefore, F-box protein is essential in the regulation of SCF activity. So far, studies of Fbxw7 have been mainly focused on its tumor suppression. This study provided an intriguing pathway in which phosphorylation-stimulated, ubiquitin-dependent degradation of target protein comprises a critical loop of determining neuronal survival and death. This study is quite in line with our study demonstrating a novel cell-death axis of Cdk5-Fbxw7-c-Jun in which Cdk5-stimulated phosphorylation of Fbxw7 contributes to regulation of cell death by causing accumulation of its substrate c-Jun during excitotoxicity. This agreement of results demonstrates that the E3 ligase are key player in determining neuronal death in experimental models of excitotoxicity^51^. Taken together, these data support a notion that the ubiquitin E3 ligase pathway might be regarded as a potentially novel and critical component in the regulation of neurodegeneration.

Previously it has been demonstrated that oxidative stress-activated Plk2 act as a negative regulator of Fbxw7^52^. Our study provides evidence that Cdk5/p25 plays the role of another negative regulator of Fbxw7. More specifically, phosphorylation of Fbxw7 by Cdk5/p25 is a novel mechanism for the control of Fbxw7 protein stability. Furthermore, Cdk5/p25 hyper-activity leads to neuronal death via decreased Fbxw7 level and concomitant accumulation of c-Jun. Here, we addressed the idea that homeostatic maintenance of Fbxw7 protein level is not limited to tumorigenesis. Our findings allow us to speculate that with proper regulation, Fbxw7 is a potential conceivable therapeutic concept for mitigation of neuronal death^53^.

## Materials and methods

### Plasmid preparation

GST-tagged human Fbxw7 was generated by sub-cloning into the pGEX4T-3 bacterial expression vector. Flag, V5, HA, and Myc sequences were integrated into the *Nhe1* site of a pCI-neo mammalian expression vector (Promega, Madision, WI). Flag-tagged human Fbxw7 were sub-cloned into the pCI-neo vector. Multiple Fbxw7 mutants were generated using a site-directed mutagenesis kit (Agilent Technologies, Inc., Santa Clara, CA). Generation of V5-, and HA-tagged wild-type (WT) mouse Cdk5 and dominant-negative Cdk5 (DN), as well as HA- and Myc-tagged mouse p25 and p35 were described previously^30,54^. All plasmids were purified using endotoxin-free plasmid DNA isolation kits (Invitrogen, Carlsbad, CA).

### Cell culture and transfection

HEK293 and HEK293T cells were cultivated at 37 °C in Dulbecco’s Modified Eagle’s Medium supplemented with 10% heat-inactivated fetal bovine serum (FBS; WelGene, Gyeongsan, Republic of Korea) in an atmosphere of 95% air and 5% CO_2_. For transient expression, HEK293 and HEK293T cells were transfected with a pre-determined amount of the indicated DNA using polyethylenimine (Sigma–Aldrich, St. Louis, MO). Establishment of primary cultures of cortical neurons was accomplished as previously described^55^. Briefly, primary cortical neurons were isolated from E14.5 mouse embryos. Dissociated cortical cells were treated with 1 mg/ml papain (Worthington Biochemical Corp., Lakewood, NJ) at room temperature for 5 min. After enzyme inactivation, isolated neurons were homogenized in Hank’s balanced salt solution (Sigma–Aldrich) supplemented with 40 μl DNase (10 unit/μl) and passed through a 70-μm mesh cell-strainer (Fisher Scientific, Hampton, NH). The singly dispersed neurons were plated on densities of either 1.25 × 10^6^ cells per well of a 6-well plate or 7.5 × 10^6^ cells per 10-cm plate that were pre-coated with 25 μg/ml poly-D-lysine (Sigma–Aldrich) and 1 μg/ml laminin (Invitrogen). Cultures were then cultivated in neurobasal medium (Invitrogen) supplemented with 2% B27 (Invitrogen) and 0.5 mM L-glutamine (Sigma–Aldrich). Half of the medium was replaced every 3–4 days. All experiments were conducted after 11 days in vitro. All mice were handled in accordance with the Yonsei University guidelines for animal care and use. All experimental procedures were approved (2017-10-647-01 and 2018-01-689-01). When appropriate, cells were treated for the indicated time periods with an empirically determined concentration of MG132 (Enzo Life Sciences Inc., Farmingdale, NY), cycloheximide (CHX; Sigma–Aldrich), roscovitine (Sigma–Aldrich), glutamate (Sigma–Aldrich), hydrogen peroxide (Sigma–Aldrich), and MPP^+^ iodide (Sigma–Aldrich). The incubation periods of all drugs used in this study were also empirically determined.

### Fbxw7 protein production and in vitro kinase assays

GST-tagged WT and mutant variants of human Fbxw7 were expressed in BL21 bacteria with 0.1 mM isopropyl-β-D-thiogalactopyranoside (Sigma–Aldrich) and incubated at 37 °C for 4 h. Bacterial pellets were resuspended in a lysis buffer containing 30 mM Tris-HCl (pH 7.5), 0.1 mM NaCl, 1% Triton X-100 (Sigma–Aldrich), 1 mM DTT (Sigma–Aldrich), and 1 × protease inhibitor cocktail (Roche Applied Science, Mannheim, Germany) and then sonicated on ice. Lysates were centrifuged at 12,000 × *g* for 15 min at 4 °C. The resulting supernatant was incubated overnight at 4 °C with GST beads (GE Healthcare, Chicago, IL). The protein samples were washed three times with 1 × PBS. GST-bound human Fbxw7 protein was incubated with 0.1 μg Cdk5/p35 (Signalchem, Richmond, BC) or Cdk5/p25 (Millipore, Billerica, MA) in a reaction buffer containing 25 mM Tris (pH7.5), 100 mM NaCl, 1 mM DTT, 100 μM unlabeled ATP and 5 μCi [γ-32P] ATP at 30 °C for 30 min. Roscovitine (10 μM) was added 30 min prior to the kinase reaction. The samples were resolved by SDS-PAGE and visualized by autoradiography.

### Immunoblot and immunoprecipitation assays

Cell lysates were prepared using RIPA lysis buffer containing 5 mM NaF, 2 mM Na_3_VO_4_ and 1 × protease inhibitor cocktail. For detecting phosphorylation status, RIPA lysis buffer containing 25 mM NaF, 2 mM, 2 mM Na_3_VO_4_ and 1 × protease inhibitor cocktail was used. After incubation on ice for 30 min, lysates were collected by centrifugation at 12,000 × *g* at 4 °C for 15 min. The supernatants were subjected to immunoprecipitation and immunoblot analysis using the indicated antibodies. For immunoprecipitation of Flag-tagged Fbxw7, cell lysates (1 mg) were incubated with 20 μl of anti-Flag M2 affinity gel beads (Sigma–Aldrich) overnight at 4 °C. The conjugates were collected by centrifugation at 800 × *g* for 2 min at 4 °C. Proteins were eluted from the beads by adding protein sample buffer and then denatured by boiling. Protein samples were then separated by SDS-PAGE and subjected to immunoblot analysis using the indicated antibodies. Antibodies used included: horseradish peroxidase (HRP)-conjugated anti-Flag antibody (A8592, 1:5,000; Sigma–Aldrich); HRP-conjugated anti-HA (3F10, 1:10,000; Roche Applied Science); anti-Myc-tag (2276, 1:2,000; Cell Signaling Technology, Beverly, MA); HRP-conjugated anti-V5 (R961-25, 1:2,000; Invitrogen); anti-Fbxw7 (A301-720A, 1:2,000; Bethyl Laboratories); anti-GAPDH (A300-641A, 1:5,000; Bethyl Laboratories); anti-phospho-Cdk5-substrate motif (9477, 1:1 000; Cell Signaling Technology); mouse anti-Cdk5 (12134, 1:2,000; Cell Signaling Technology); rabbit anti-Cdk5 (14145, 1:2,000; Cell Signaling Technology); anti-p35/p25 (2680, 1:2,000; Cell Signaling Technology); anti-GST (sc459, 1:5,000; Santa Cruz Biotechnology, Inc. Dallas, TX); anti-c-Jun (9165, 1:2,000; Cell Signaling Technology); anti-ub (P4D1, 1:1,000, Santa Cruz Biotechnology, Inc.); HRP-conjugated goat anti-rabbit antibody (AP132P, 1:5,000; Millipore); and HRP-conjugated goat anti-mouse antibody (AP124P, 1:5,000; Millipore). Specific bands were detected with enhanced chemiluminescence (PerkinElmer, Waltham, MA).

### Endogenous binding of Fbxw7 and Cdk5 in primary cultures of cortical neurons

Primary cultures of cortical neurons were maintained until 11 days in vitro for this purpose. For immunoprecipitation of Fbxw7 and Cdk5, lysates from cortical neurons were pre-incubated with protein A agarose beads (Millipore) for pre-clearing, and then further incubated with 1 μg of anti-Fbxw7 (A301-720A, Bethyl Laboratories) and 4 μg of anti-Cdk5 (sc-6247, Santa Cruz Biotechnology, Inc.), respectively. For controls, both rabbit anti-IgG (I8140, Sigma–Aldrich) and mouse anti-IgG (I8765, Sigma–Aldrich) were used. After overnight incubation, lysates were further incubated with protein A agarose bead for conjugation. The conjugates were collected by centrifugation at 800 × *g* for 2 min at 4 °C. Proteins were eluted from the beads by adding protein sample buffer and then denatured by boiling. Protein samples were then separated by SDS-PAGE and subjected to immunoblot analysis using anti-Fbxw7 (A301-720A, 1:2,000; Bethyl Laboratories) and anti-Cdk5 (12134, 1:2,000; Cell Signaling Technology) antibodies.

### Lentivirus infection

Cdk5 knockdown for RNA interference was achieved using Mission shRNA-encoding lentivirus directed to mouse Cdk5 mRNA (Sigma–Aldrich; GenBank/EMBL/DDBJ accession no. NM_007668) as recommended by the manufacturer’s protocols. Briefly, lentiviral vectors (in pLKO.1) containing Cdk5 shRNA sequences (TRCN0000009521) and non-target shRNA control vector (shScramble, SHC016) were purchased from Sigma^30^. The lentivirus particles were generated by co-transfection into HEK293T cell with lentivirus vectors and three other vectors including pMDLg/pRRE, pMD2.G and pRSV-Rev (all from Addgene), using polyethylenimine (PEI). Two days after transfection, the cell culture media were filtered using a 0.45-μm filter (Millipore). Infection to primary neuron was conducted after 3 days in vitro. After 24 h of infection at 37 °C, the culture media was replaced with fresh culture media. Cdk5 protein level was assessed by immunoblot analysis. All experimental procedures were approved by the Institutional Biosafety Committee of Yonsei University (IBC-A-201901-192-03).

### RNA Extraction and quantitative real-time reverse transcriptase-polymerase chain reaction analysis

Total RNA was prepared using an RNAeasy micro kit (Qiagen) and cDNA was generated using a SuperScriptIII First-Strand System (Fisher Scientific) according to the manufacturer’s instructions. Quantitative real-time PCR analyses were performed using a Power SYBR^TM^ Green PCR Master Mix (Fisher Scientific) with the CFX 384 Touch Real-Time PCR Detection System (Bio-Rad). To analyze human Fbxw7 mRNA expression levels, the following primer pairs were used; human Fbxw7, 5′-AAAGAGTTGTTAGCGGTTCTCG-3′ and ′5-CCACATGGATACCATCAAACTG-3′; human GAPDH, 5′-TGAACCACCAACTGCTTAGC-3′ and 5′-GGCATGGACTGTGGTCATGAG-3′.

### Statistical analysis

Data are expressed as the mean ± standard deviation (SD) for three independent experiments. To determine the significance of differences between groups, two-tailed Student’s *t*-tests or one-way analysis of variance (ANOVA) followed by Tukey’s post-hoc tests were performed using Prism 6 software (GraphPad Inc., San Diego, CA). Statistical significance of differences is indicated as follows: ^***^p < 0.001, ^**^p < 0.01, and ^*^p < 0.05.

## Supplementary information

Supplementary informations
