# Supplementary material for: Site-specific phosphorylation of Fbxw7 by Cdk5/p25 and its resulting decreased stability are linked to glutamate-induced excitotoxicity
Source: Cell Death Dis. 2019 Aug 2;10(8):579. doi: 10.1038/s41419-019-1818-4 (PMC6675790; doi:10.1038/s41419-019-1818-4)
Supplement: Supplementary file 1 — Supplementary informations [file 41419_2019_1818_MOESM1_ESM.pdf]

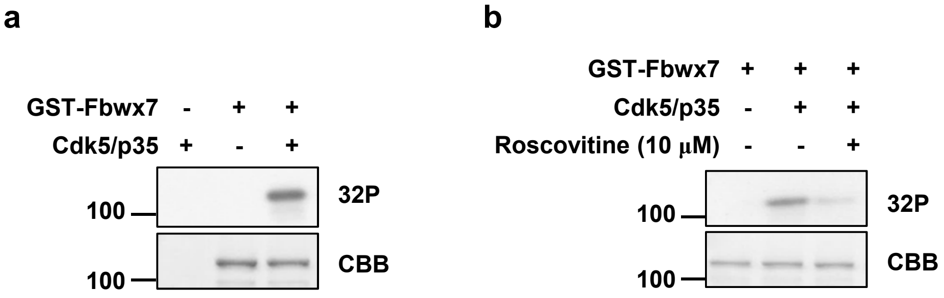

Supplementary Figure 1. Cdk5/p35 phosphorylates Fbxw7. (a) *In vitro* kinase assay for detecting phosphorylation of Fbxw7 by Cdk5/p35. Purified GST-Fbxw7 protein (1 μg) was incubated with or without 0.1 μg recombinant GST-Cdk5/GST-p25 protein in the presence of [γ-32P] ATP. **(b)** To inhibit Cdk5 kinase activity, samples were treated with 10 μM roscovitine for 30 min before reaction. Reaction mixtures were resolved by SDS-PAGE and visualized by autoradiography. Coomassie brilliant blue (CBB) staining for GST-Fbxw7 protein was used as a loading control.

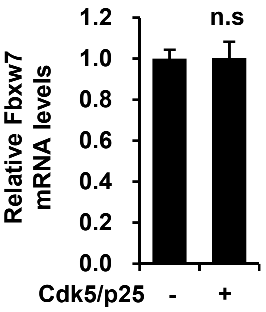

Supplementary Figure 2. Overexpression of Cdk5/p25 dose not affect mRNA levels of Fbxw7. HEK293T cells were transfected with vector containing the sequences with or without Cdk5/p25. Total RNA were extracted and subjected to qRT-PCR. Data were normalized to the internal control (GAPDH) and presented as a relative expression level over the control (value = 1). Bar represents the mean ± SD from four independent experiments. n.s, not significant

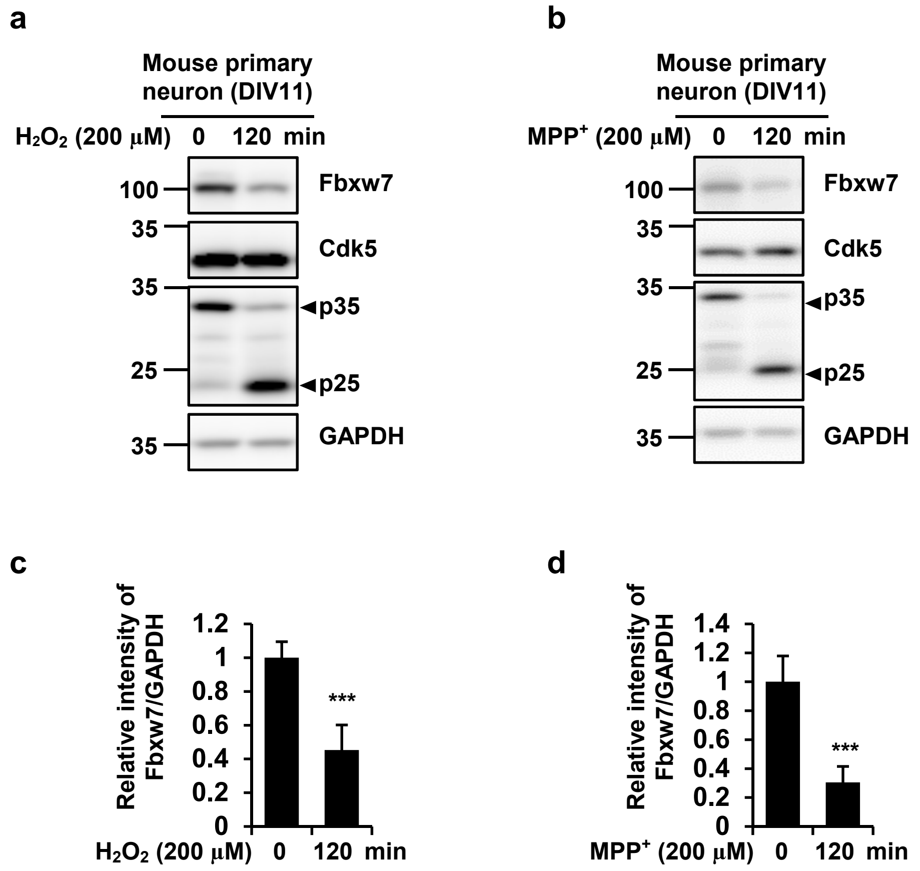

Supplementary Figure 3. Decreased expression of Fbxw7 is detected in other pathological conditions. At DIV11, primary cultures of cortical neurons were treated with **(a)** 200 μM hydrogen peroxide (H_2O_2) for 120 min and **(b)** 200 μM MPP^+ iodide (MPP^+) for 120min, respectively. Cell lysates were harvested and analyzed by IB using the indicated antibodies. **(c, d)** Relative fold intensity of Fbxw7 was quantified over the non-treated control (value = 1). Bar represents the mean ± SD from three independent experiments. *** $p<0.001$.
